# Lower‐limb muscle responses evoked with noisy vibrotactile foot sole stimulation

**DOI:** 10.14814/phy2.14530

**Published:** 2020-08-09

**Authors:** Ryan M. Peters, Robyn L. Mildren, Aimee J. Hill, Mark G. Carpenter, Jean‐Sébastien Blouin, J. Timothy Inglis

**Affiliations:** ^1^ Faculty of Kinesiology University of Calgary Calgary AB Canada; ^2^ School of Kinesiology University of British Columbia Vancouver BC Canada; ^3^ Djavad Mowafaghian Centre for Brain Health University of British Columbia Vancouver BC Canada; ^4^ International Collaboration on Repair Discoveries University of British Columbia Vancouver BC Canada; ^5^ Institute for Computing, Information, and Cognitive Systems University of British Columbia Vancouver BC Canada

## Abstract

**Aim:**

Cutaneous feedback from the foot sole contributes to the control of standing balance in two ways: it provides perceptual awareness of tactile perturbations at the interface with the ground (e.g., shifts in the pressure distribution, slips, etc.) and it reflexively activates lower‐motor neurons to trigger stabilizing postural responses. Here we focus on the latter, cutaneous (or cutaneomotor) reflex coupling in the lower limb. These reflexes have been studied most‐frequently with electrical pulse trains that bypass natural cutaneous mechanotransduction, stimulating cutaneous afferents in a largely non‐physiological manner. Harnessing the mechanical filtering properties of cutaneous afferents, we take a novel mechanical approach by applying supra‐threshold continuous noisy vibrotactile stimulation (NVS) to the medial forefoot.

**Methods:**

Using NVS, we characterized the time and frequency domain properties of cutaneomotor reflexes in the Tibialis Anterior. We additionally measured stimulus‐triggered average muscle responses to repeated discrete sinusoidal pulses for comparison. To investigate cutaneomotor reflex gain scaling, stimuli were delivered at 3‐ or 10‐times perceptual threshold (PT), while participants held 12.5% or 25% of maximum voluntary contraction (MVC).

**Results:**

Peak responses in the time domain were observed at lags reflecting transmission delay through a polysynaptic reflex pathway (~90–100 ms). Increasing the stimulus amplitude enhanced cutaneomotor coupling, likely by increasing afferent firing rates. Although greater background muscle contraction increased the overall amplitude of the evoked responses, it did not increase the proportion of the muscle response attributable to cutaneous input.

**Conclusion:**

Taken together, our findings support the use of NVS as a novel tool for probing the physiological properties of cutaneomotor reflex pathways.

## Graphical abstract

Here we describe a novel approach for measuring cutaneous reflex function using noisy vibrotactile stimulation (NVS) of the skin. We examine the effects of changing the stimulus amplitude and background contraction level on the evoked responses. Taken together, our findings support the use of NVS as a powerful new tool for probing the physiological properties of cutaneomotor reflex pathways.

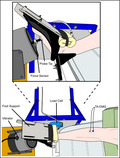

## 1 INTRODUCTION

Information originating from cutaneous receptors of the foot sole is thought to be one of the multiple sensory inputs that contribute to human balance control (Inglis, Kennedy, Wells, & Chua, 2002; Kennedy & Inglis, 2002). Dynamic shifts in the plantar pressure distribution (Kavounoudias, Roll, & Roll, 1998, 1999) or sudden foot slip events (Johannson & Westling, 1984; Macefield, Hägger‐Ross, & Johansson, 1996; Srinivasan, Whitehouse, & LaMotte, 1990) are examples of balance‐relevant tactile events encoded by plantar cutaneous receptors. The importance of cutaneous information in standing balance is also suggested by the increase in postural sway that has been observed in young adults when plantar skin input is reduced experimentally, either by anesthesia (Meyer, Oddsson, and De Luca, 2004)) or cooling the skin of the foot sole (Magnusson, Enbom, Johansson, & Wiklund, 1990; Perry, McIlroy, & Maki, 2000). Previous studies have demonstrated that vibration applied to particular regions of the foot sole (e.g., heel vs. forefoot) results in patterned muscle (Zehr et al., 2014) and whole‐body balance responses (Kavounoudias, Roll, & Roll, 1998, 1999). More specifically, these studies demonstrate that vibrating both heels results in individuals swaying forward, vibrating the forefoot bilaterally results in individuals swaying backward, and vibrating one heel or forefoot alone results in diagonally directed sway. This close correspondence between the site of plantar vibration and whole‐body balance responses has led researchers to suggest that foot sole cutaneous input provides a “dynamometric map” for controlling standing balance (Kavounoudias et al., 1998, 1999). Cutaneous‐evoked postural responses are believed to arise through spinal, and potentially, transcortical reflex pathways that couple cutaneous afferent input to lower motor neuron activity. *Cutaneomotor coupling* of this sort has previously been established on the single‐unit level via human microneurography experiments (Bent & Lowrey, 2013; Fallon, Bent, McNulty, & Macefield, 2005).

Researchers have deployed a wide range of skin stimulation techniques to evoke cutaneous reflexes and postural responses, including electrical pulses (Aniss, Gandevia, & Burke, 1992; Zehr et al., 2014), manual stroking and blowing (Bent & Lowrey, 2013; Fallon et al., 2005), discrete skin indentations (Forth & Layne, 2007; Pang & Yang, 2000), and bursts of sinusoidal skin vibration (Duysens, Beerepoot, Veltink, Weerdesteyn, & Smits‐Engelsman, 2008). Notably, mechanical stimulation offers particular advantages. Similar to electrical stimulation, skin vibration can be precisely controlled in terms of frequency, amplitude, and duration. In contrast to electrical stimulation, however, mechanical stimuli activate cutaneous receptors via natural mechanotransduction processes. Mechanotransduction in the four cutaneous afferent classes is selective for particular, albeit overlapping, bandwidths of vibration frequencies (Bolanowski, Gescheider, Verrillo, & Checkosky, 1988; Johansson & Vallbo, 1979; Johnson, 2001; Johnson, Yoshioka, & Vega‐Bermudez, 2000; Kennedy & Inglis, 2002; Löfvenberg & Johansson, 1984; Strzalkowski, Ayesha, & Bent, 2017). Therefore, by manipulating the bandwidth of stimulation, the afferent population response can presumably be biased toward different afferent classes, providing a window into their relative contribution to cutaneomotor reflexes (Bent & Lowrey, 2013; Duysens et al., 2008; Fallon et al., 2005; Peters, McKeown, Carpenter, & Inglis, 2016).

To build upon previous studies that used discrete stimulation (Duysens et al., 2008; Forth & Layne, 2007; Pang & Yang, 2000), here we develop continuous Noisy Vibrotactile Stimulation (NVS) as a novel means of assessing cutaneous‐evoked muscle responses over a range of vibration frequencies. Noisy stimuli have been applied to probe vestibular‐evoked reflexes (Dakin, Luu, Van Den Doel, Inglis, & Blouin, 2010; Dakin, Son, Inglis, & Blouin, 2007) and tendon vibration‐evoked reflexes (Mildren et al., 2017, 2019) in humans previously. NVS has multiple advantages over discrete stimuli. By removing temporal gaps between discrete stimulus presentations, experiment efficiency is enhanced (i.e., greater response signal‐to‐noise ratio in less time (Dakin et al., 2007; Reynolds, 2011). Also, broadband continuous noisy stimulation lends itself well to a wide range of linear systems identification techniques, providing a more complete picture of the time‐frequency relationship between stimulus and response. We recorded surface EMG to characterize the global muscle response from an ensemble of Tibialis Anterior motor units during mechanical foot sole stimulation. Cutaneomotor reflexes were evoked at two stimulus amplitudes relative to each participant's Perceptual Threshold (PT) and two percentages of Maximal Voluntary Contraction (MVC) to assess gain scaling of the muscle response. We predicted that reflex responses would be observed at polysynaptic reflex latencies of 50–100 ms (Aniss et al., 1992; Duysens et al., 2008; Forth & Layne, 2007). Because human foot sole cutaneous afferent firing rates increase along with stimulus amplitude (Strzalkowski et al., 2017), we predicted that increasing stimulus amplitude would enhance cutaneomotor coupling strength. As voluntary background EMG increases, however, we expected that the TA motor neuron pool would be driven increasingly by descending commands rather than the input cutaneous stimulus (Arntz et al., 2019). Therefore, we predicted cutaneomotor coupling would remain the same or decrease slightly at higher levels of background EMG due to increased competing synaptic input to the motor neuron pool at the spinal level.

## 2 EXPERIMENTAL PROCEDURES

### 2.1 Participants

Ten young adults (6 men, 4 women) between the ages of 22 and 30 (mean: 25.5 years, *SD*: ±2.6 years) were included in this study. We excluded participants with a history of neurological or musculoskeletal disease/injury, based on self‐report. Data from one participant for a single experimental condition using discrete pulses (25% MVC, 3 PT) are missing due to a technical issue during data collection. Informed written consent was obtained prior to each experiment. All Experimental procedures conformed to the standards of the World Medical Association Declaration of Helsinki and were approved by the University of British Columbia's Clinical Research Ethics Board.

### 2.2 Apparatus and stimuli

**FIGURE 1:**
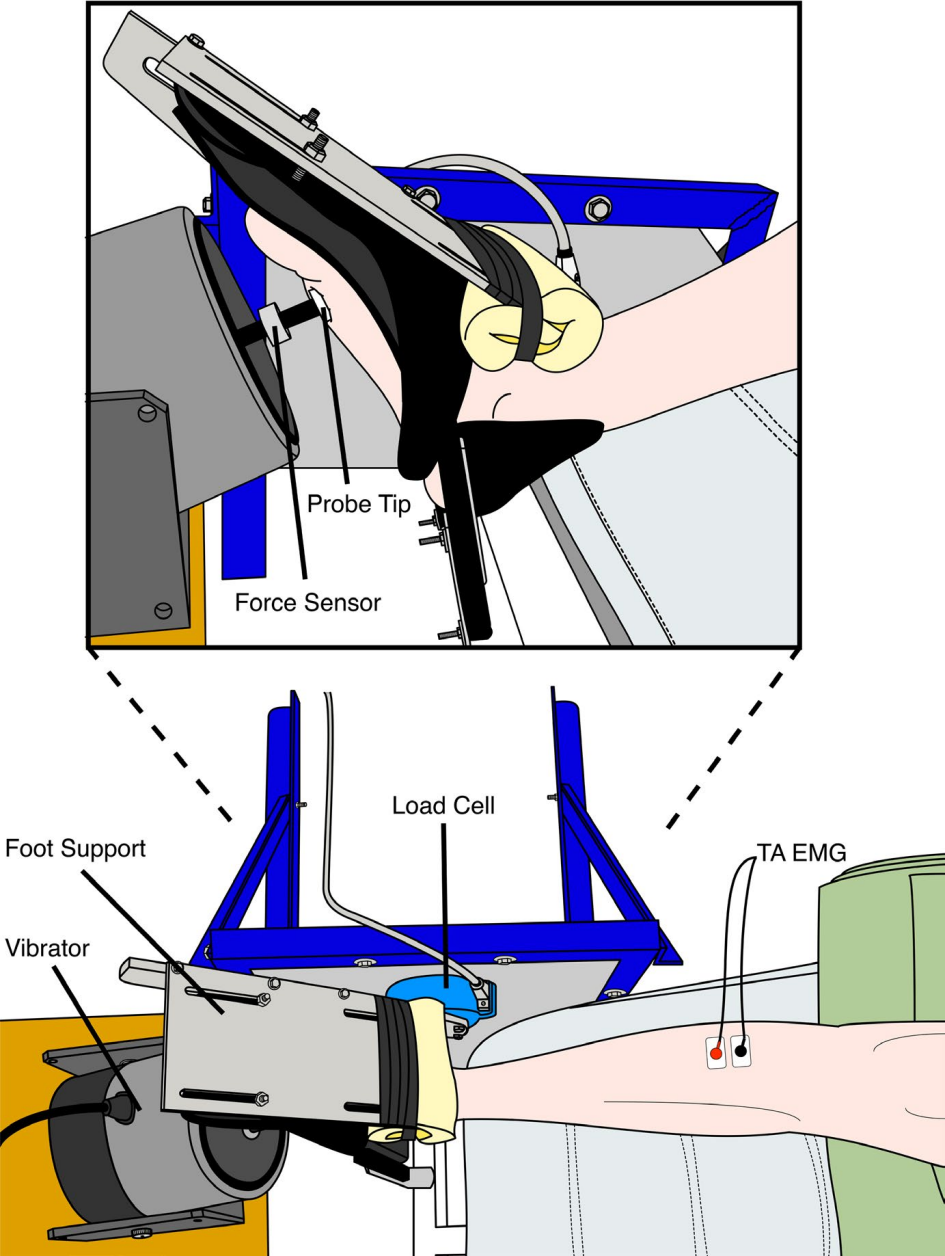
Experimental setup. Participants were seated in an adjustable chair with their right foot in a custom‐built support that was coupled with a six‐axis load cell. Participants were instructed to maintain one of the two levels of isometric dorsiflexion (12.5% or 25% of MVC) against the device using real‐time visual feedback of their ankle torque displayed on a flat‐screen television positioned 1.5 m in front of them. The probe tip of the vibrator, in‐series with a force sensor, was pressed into the skin overlaying the first metatarsal head with a ~5 N preload prior to delivering vibration. Surface EMG was recorded from the participant's right Tibialis Anterior. Inset: side view of apparatus

Participants were seated in a comfortable chair, with their right foot resting in a custom‐built dorsiflexion monitoring apparatus, as depicted in Figure 1. The heel was held firmly with ankle support, and the dorsum of the foot was held flush against a plastic form fixed to an aluminum plate linked to a 6‐axis load cell (JR31000N125, Multi‐Axis Load Cell Technologies), which was positioned such that the z‐axis was collinear with the ankle joint (see Figure 1). This setup allowed for continuous monitoring of the isometric ankle dorsiflexion torque produced by the participant, and given this, we chose to measure and analyze muscle activity from Tibialis Anterior (see below). Ankle torque was digitized at 1 kHz using an A/D board (Power 1401, Cambridge Electronic Design), and controlled by Spike2.0 software (Cambridge Electronic Design). Prior to reflex testing, participants performed two maximal voluntary contractions (MVC) with verbal encouragement. A one‐minute rest period was provided between contractions. We based the ankle torque level during reflex testing (12.5% or 25% of MVC) on the peak value observed across attempts. MVC levels were chosen to make our results comparable to previous work (Forth & Layne, 2007). We supplied participants with biofeedback on their ongoing ankle torque level, along with a line indicating the target level via Spike2.0 displayed on a flat panel television positioned 1.5 m in front of the subject. Knee angle was supported at approximately 110° during all testing procedures.

**FIGURE 2:**
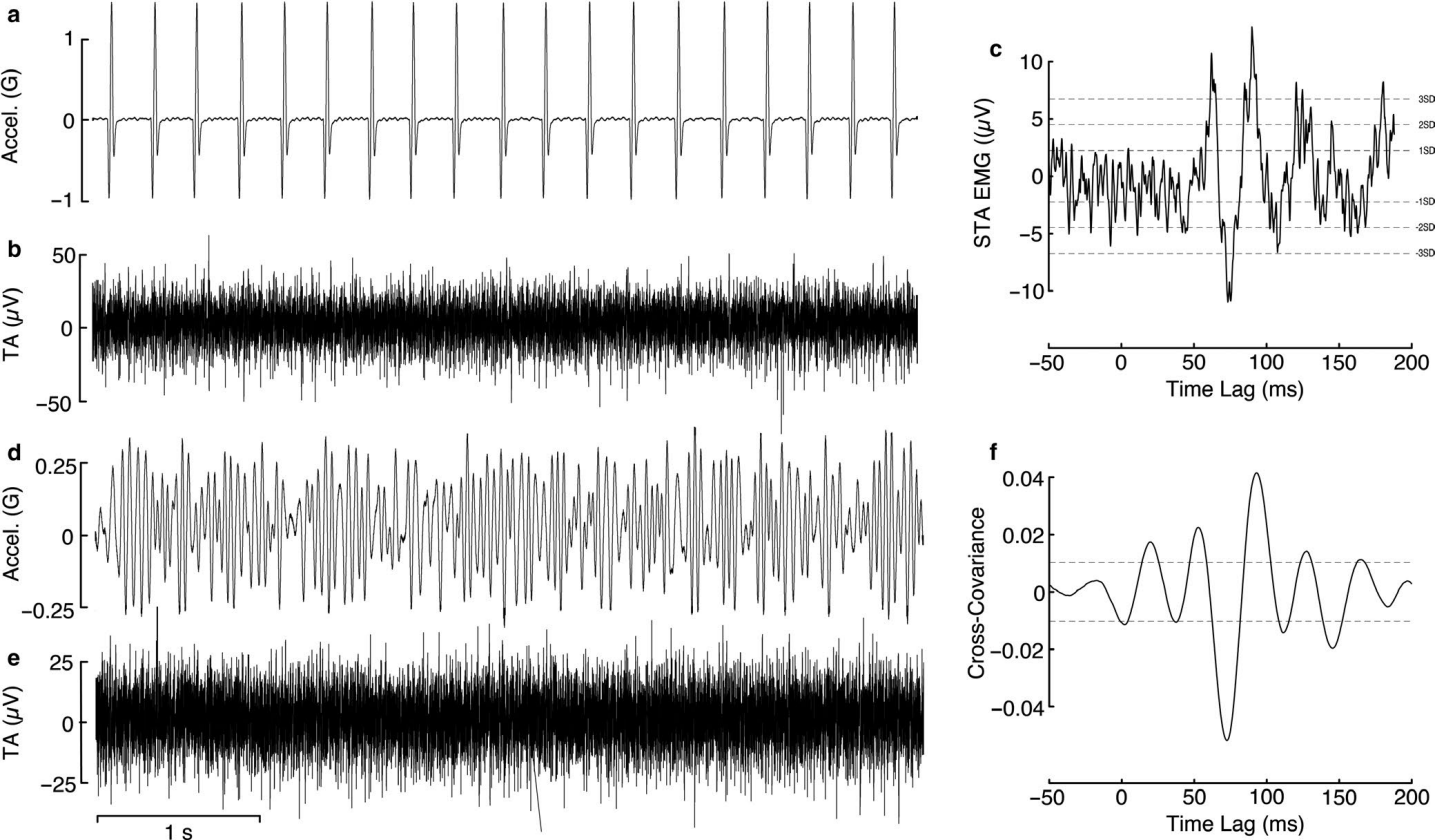
Sample data from a representative participant using both discrete pulses and NVS. (A) Discrete pulse stimulus acceleration profile and (B) raw EMG over a 5‐s interval taken from a sequence of 600 pulses with the stimulus amplitude set to 10 PT and the background level of contraction held at 12.5% MVC. (C) Stimulus‐triggered average EMG trace for the corresponding trial. Stimulation resulted in an oscillatory temporal EMG pattern on average with a peak at 90.7 ms and trough at 73.7 ms. Horizontal dashed lines denote the 1, 2, and 3 *SD* of the background EMG, calculated as the *SD* of the background EMG in the 50 ms preceding *t* = 0. (D) NVS acceleration profile and (E) raw EMG over a 5‐s interval taken from a 120‐s trial with the stimulus amplitude set to 10 PT and the background level of contraction held at 12.5% MVC. (F) Cross‐covariance between NVS and surface EMG for the corresponding trial. Horizontal dashed lines denote the 95% confidence interval level for statistical significance. The cross‐correlation function has an oscillatory temporal correlation pattern with a peak at 93 ms and a trough at 72.6 ms

Tactile stimuli were delivered using a linear shaker (MT‐160, Labworks Inc.), that was equipped with a contact force sensor (Model 31, Honeywell), and an accelerometer (220‐010, X Tronics) positioned in‐series with a 3‐cm diameter acetyl plastic probe‐tip. With the participant holding the desired torque level, the vibrator was pre‐loaded against the medial forefoot at the base of the first toe with 5 N of contact force, which was enough to keep firm contact between the skin and probe tip during the trials. The medial forefoot was chosen as the stimulation site because it is predicted to have an excitatory input onto Tibialis Anterior based on studies that used electrical stimulation (Zehr et al., 2014) and has been linked to balance control mechanisms in previous research (Cruz‐Almeida, Black, Christou, & Clark, 2014). Before testing, each participant's perceptual threshold (1 PT) was estimated using the method of adjustment (Wells, Ward, Chua, & Inglis, 2003). Briefly, the stimulus amplitude was manually increased until the participant could just reliably detect the tactile stimulation and then decreased until they lost this sensation, in an alternating ascending/descending series. PT was set to the average amplitude across three ascending and descending series where the participant transitioned from detecting to not detecting the vibration. Repeating this procedure, we estimated PT values for both NVS and discrete pulses. Acceleration and force signals were differentially amplified, analog low‐pass filtered at 600 Hz (Brownlee model 440, AutoMate Scientific Inc.), and then digitized at 5 kHz (via Power 1401 board) into Spike2.0 (Cambridge Electronic Design). The signal driving the linear shaker was generated using LabVIEW 11 (National Instruments) and output as an analog voltage signal at 5 kHz from multifunctional data acquisition card (PXI‐6225, National Instruments), controlled by a real‐time computer (PXI‐8106, National Instruments). Analog voltage commands were sent to a motor amplifier (PA‐141, Labworks Inc.) for open‐loop control of the vibrotactile stimulation. NVS signals were low‐pass filtered white noise waveforms, with a low‐pass cut‐off frequency of 30 Hz. Continuous 120s trials were collected for each test condition. Figure 2 depicts the resulting acceleration power spectrum, which contains frequencies up to 40 Hz. Discrete sinusoidal pulse stimuli were delivered by driving the motor with 30 Hz raised cosine bell curve waveforms delivered at randomized inter‐stimulus intervals (160–200 ms) for a total of 600 pulses total per condition.

### 2.3 Surface electromyography

Surface electromyography (EMG) was recorded from the right tibialis anterior (TA) muscle using surface electrodes positioned over the shaved and cleaned muscle belly in the bipolar arrangement (oriented as in Figure 1). A surface ground electrode was placed over the right lateral malleolus. Surface EMG was amplified ×2000 and band‐pass filtered from 0.01 to 10 kHz by a pre‐amplifier (NeuroLog NL824, Digitimer Ltd.) and isolator (NeuroLog NL820, Digitimer Ltd.). Surface EMG was digitized at 5 kHz with a Power 1401 AD board (Cambridge Electronic Design). Recordings were monitored real‐time in Spike2.0 along with the ankle torque, contact force, and acceleration signals. Surface EMG was band‐pass filtered between 0.01 and 10 kHz (4th order dual‐pass Butterworth) and full‐wave rectified offline in MATLAB 2019a (Mathworks).

### 2.4 Data analysis

We compared temporal and spectral aspects of cutaneomotor muscle responses across different levels of stimulus amplitude and background contraction. In the time domain, we computed the Peak‐to‐Peak (P2P) amplitude and latency of either the cross‐covariance function (NVS) or stimulus‐triggered average EMG (discrete pulses). In the frequency domain, we computed pooled estimates of coherence, gain, and phase spectra (NVS only) across all subjects. We used the NeuroSpec2.0 software package for MATLAB (Halliday et al., 1995; Rosenberg, Amjad, Breeze, Brillinger, & Halliday, 1989) to compute all cross‐covariance, coherence, gain, and phase estimates. Stimulus‐triggered averages were computed with Spike2.0 (Cambridge Electronics Design).

To identify temporal characteristics of the cutaneomotor input‐output relationship during NVS, cross‐covariance functions were calculated as the inverse Fourier transform of the input‐output cross‐spectra (Halliday et al., 1995), normalized by the product of the vector norms of the input and output signals (Dakin et al., 2010). Through this additional normalization step, the cross‐covariance values become bounded between −1 and 1. Our convention was that acceleration toward the skin and increased (rectified) EMG were assigned positive polarities. Within this convention, a positive correlation would represent positive accelerations being associated with increased EMG or negative accelerations being associated with decreased EMG. We note that this convention is arbitrary, but consistent with previous research on noisy tendon vibration, where positive polarities were defined as “into the tendon” (Mildren et al., 2017, 2019). Velocity and position signals could also be represented by sinusoidal waves at the same frequency but with their phase shifted by 90 degrees (for velocity) or 180 degrees (for position) and amplitude modulated in a frequency‐dependent manner. Therefore, the results from the analyses presented in the paper would be similar if we used a velocity or position input signal, but with mathematically predictable changes in the phase and gain relationships between the input and output signals. Cross‐covariance estimates were constructed for each subject, as well as on a pooled basis by concatenating individual subject data together for each of the four unique stimulus amplitude/background contraction level test conditions. Data were sub‐divided into non‐overlapping Fast Fourier Transform (FFT) segments that were 2 (Fallon et al., 2005) samples each (0.8192 s in duration), providing a frequency resolution of 1.2207 Hz. For individual subjects, each 120 s trial was clipped to be 598,016 samples (i.e., exactly 146 FFT segments). Pooled data for each of the four unique test conditions were generated by concatenating each participant's 146 non‐overlapping FFT segments together, resulting in a total of 1,460 FFT segments in subsequent pooled analyses (*N* = 10 subjects). The 95% confidence limits for coherence (positive threshold) and cross‐covariance (positive and negative thresholds) were constructed under the hypothesis of independence between the two signals.( Halliday et al., 1995) Values exceeding these limits provide evidence of a significant linear relationship between the stimulus and response. For the discrete pulses, stimulus‐triggered averages were computed using Spike2.0 and corrected for the 11.3 ms lag between the digital trigger event and the peak of the resulting acceleration pulse. We considered responses significant when the peak exceeded ± 1 *SD* of the averaged background EMG, calculated from the 50 ms preceding the stimulus. We report both raw and normalized stimulus‐triggered average (STA) P2P amplitudes. Raw STAs were constructed to make our findings compared to previous studies that did not normalize their stimulus‐triggered averages to background EMG (Duysens et al., 2008; Forth & Layne, 2007). We also normalized the STAs to make them comparable to the coherence/cross‐covariance results for NVS, as these latter quantities are both normalized to the background EMG. Normalization to the background EMG level allows the proportion of muscle responses driven by the input stimulus to be ascertained (Reynolds, 2011). We normalized STAs by dividing the peak and trough of the response by the average standard deviation of the background EMG in 50 ms preceding the stimulus. For stimulus‐triggered averages and cross‐covariance functions, the latency of the P2P amplitude was the timepoint midway between peak and trough latencies.

To identify spectral relationships between NVS and muscle responses, we constructed pooled coherence, gain, and phase estimates. Pooled frequency domain plots for each of the four unique test conditions were generated using the same between‐subject concatenation process described above for pooled cross‐covariance estimates. Coherence values provide an estimate of the linear correlation between two signals across frequencies and were calculated as the magnitude of the input‐output signal cross spectra squared divided by the product of the input and output auto‐spectra. Coherence was deemed to be significant if it surpassed the 95% confidence limit (Cruz‐Almeida et al., 2014; Dakin et al., 2007, 2010; Mildren et al., 2017, 2019). For frequencies exhibiting significant coherence, we additionally generated pooled gain and phase plots describing the input‐output relationship.

### 2.5 Statistical analysis

To compare the time‐domain data (P2P amplitudes and latencies) across the different test conditions, we performed repeated measures ANOVAs with stimulus amplitude (3 and 10 PT) and background contraction level (12.5% and 25% MVC) as the within‐subject factors. Post‐hoc pairwise comparisons were performed based on the estimated marginal means from the four different test conditions and Bonferroni correction was performed to adjust for multiple comparisons (adjusted α level of 0.0125). To compare peak response latencies obtained using the two different stimuli and analysis methods (NVS vs. discrete pulses), we performed a paired‐samples *t*‐test on the observed P2P latencies using the two different methods. P2P response amplitudes were not statistically compared due to differences in the units and normalization procedures used in the discrete vs. continuous analysis approaches. Data analysis was carried out with custom MATLAB scripts to extract dependent measures and SPSS v26 (IBM Corporation) to perform statistical testing. In all cases, an α level of 0.05 was used to ascertain statistical significance.

## 3 RESULTS

### 3.1 NVS‐evoked muscle responses in TA

**FIGURE 3:**
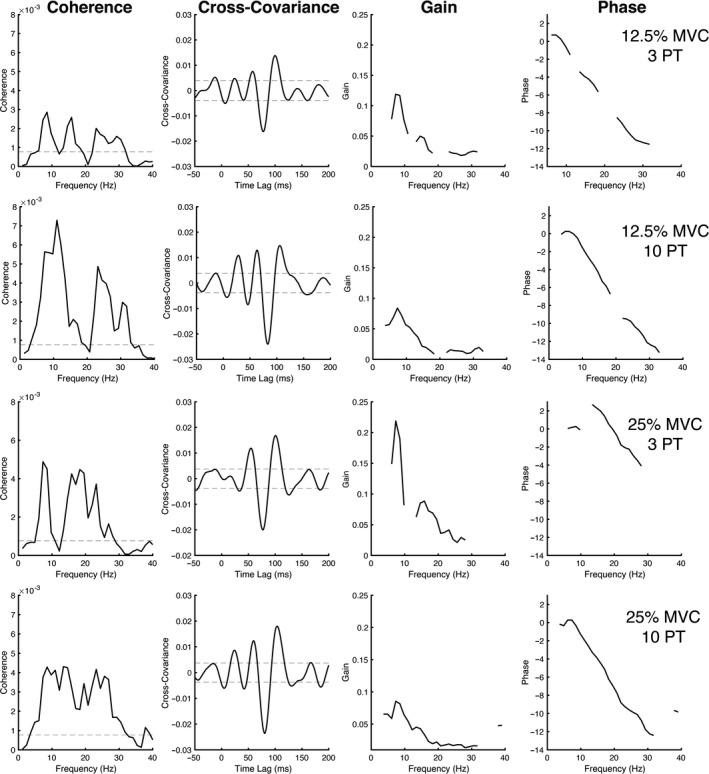
Pooled data for the different stimulus amplitudes and background levels of contraction (*N* = 10 subjects). Horizontal lines indicate the level of statistical significance for the coherence spectra and cross‐covariance plots. Pooled gain and phase estimates are also displayed for frequencies exhibiting significant coherence. The bandwidth of coherence was significant within the 5–30 Hz range, which coincides with the frequency bandwidth of the NVS stimulation. Gain decreased and the phase lag became more pronounced at higher frequencies

**FIGURE 4:**
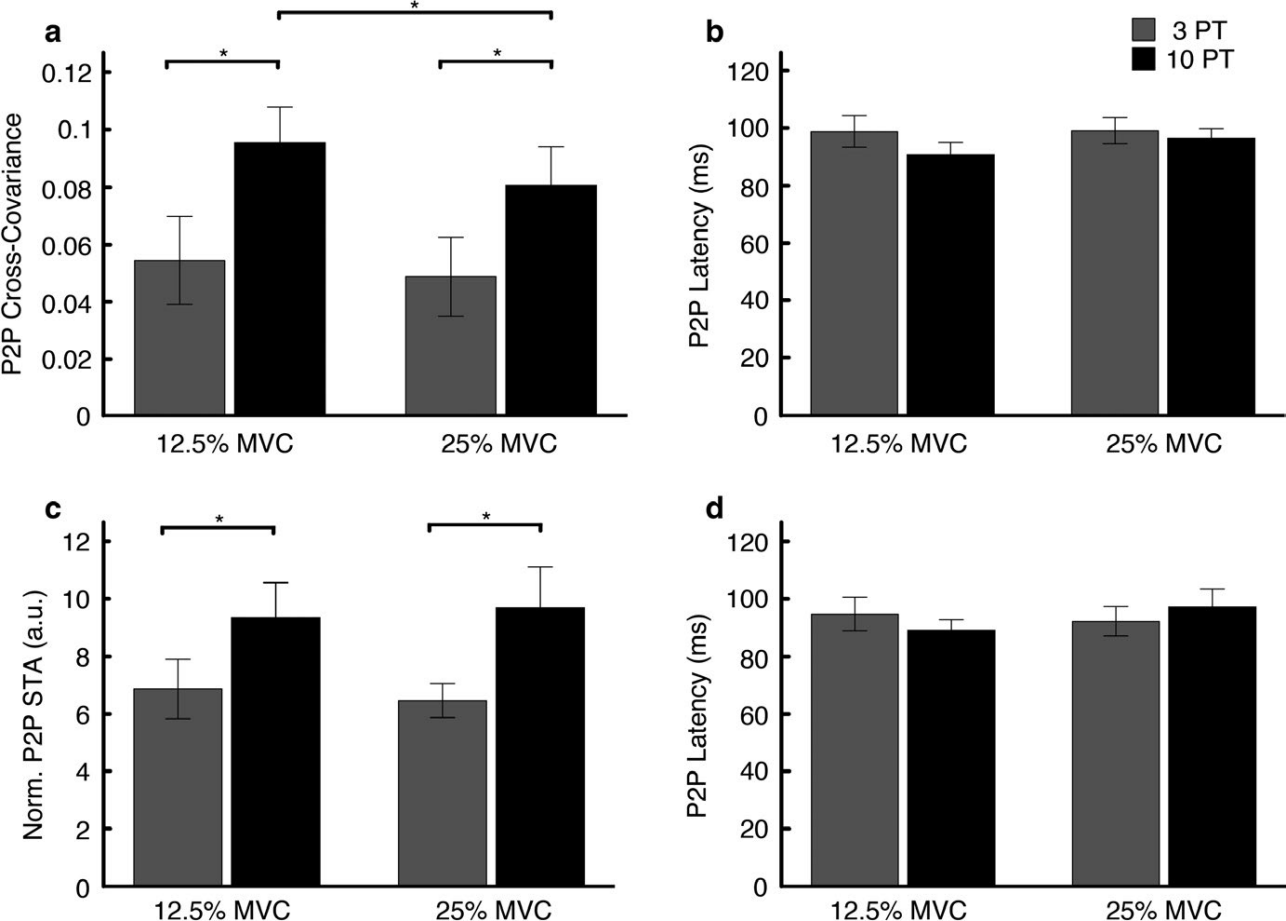
Group averages for the different stimulus amplitudes and background levels of contraction (*N* = 10 subjects). The top row of bar graphs depicts the data obtained using NVS. (A) Average Peak‐to‐Peak (P2P) cross‐covariance amplitude and (B) average cross‐covariance P2P latency. The bottom row of bar graphs depicts the data obtained using repeated tap stimuli. (C) Average P2P stimulus‐triggered average (STA) amplitude and (D) average STA P2P latency. Error bars denote ±1 *SE*

First, we examined the relationship between the 0–30 Hz NVS input stimulus and the activity of multiple motor units innervating TA. Across all participants and conditions, the response latency, as estimated by the peak in the cross‐covariance plots, occurred at a time lag of 96.3 ms (*SE* = ±2.7 ms). A 5‐s sample of raw data from a representative participant are displayed in Figure 2, as well as the cross‐covariance plots from the corresponding 120‐s trial. For reference, Figure 2 also shows data from the same participant and test condition using the STA approach (see below). Figure 3 displays the pooled coherence, cross‐covariance, gain, and phase plots for each condition, illustrating the effect of increasing stimulus amplitude and background muscle activation level on NVS‐evoked cutaneomotor response amplitude and timing. P2P cross‐covariance amplitude decreased with higher background EMG (*F*_1,9_ = 8.262, *p* = .018) but it increased with higher stimulus amplitude (*F*_1,9_ = 21.730, *p* = .001). There was no interaction between background EMG and stimulus amplitude (*F*_1,9_ = 0.899, *p* = .368). Post‐hoc comparisons revealed that P2P values were significantly higher at 10 PT (*p* = .001), and significantly lower at 25% MVC (*p* = .018). The latency of the P2P cross‐covariance was not affected by background EMG level (*F*_1,9_ = 0.888, *p* = .371) or stimulus amplitude (*F*_1,9_ = 1.314, *p* = .281), and there was no significant interaction between these factors (*F*_1,9_ = 0.372, *p* = .557). The top row of bar graphs in Figure 4 depicts the group average P2P cross‐covariance amplitude and latency across the different test conditions.

### 3.2 Comparison between discrete pulse stimuli and NVS

Next, we compared the results obtained using NVS to those obtained delivering discrete stimuli and averaging the EMG off each stimulus event (i.e., stimulus‐triggered average; STA). Data from a representative participant are shown in Figure 2. Responses consistently exhibited an oscillatory appearance, regardless of the stimulus type. Across all conditions, the latency of the P2P cross‐covariance was 93 ms (*SE* = ±3.1 ms) on average. Based on a paired‐samples *t*‐test across all participants and test conditions, the latencies of P2P responses using discrete pulses were not different from those estimated with NVS (*t*_38_ = −0.684, *p* = .498).

To determine if stimulus amplitude and background EMG had an effect on response amplitude or timing using single pulses, we performed repeated measures ANOVAs on P2P STA amplitudes and latencies. Normalized P2P STA amplitudes were not affected by increasing background EMG (*F*_1,8_ = 0.006, *p* = .942), but did increase significantly at the higher stimulus amplitude (*F*_1,8_ = 14.369, *p* = .005). Post‐hoc comparisons revealed that P2P values were significantly higher at 10 PT (*p* = .005). There was no interaction between background EMG level and stimulus amplitude (*F*_1,8_ = 0.441, *p* = .525). Using the non‐normalized STA values, increasing background EMG (*F*_1,8_ = 14.998, *p* = .005) and stimulus amplitude (*F*_1,8_ = 16.159, *p* = .004) both resulted in significantly larger P2P amplitudes with no interaction between these factors (*F*_1,8_ = 0.955, *p* = .357). In this case, post‐hoc comparisons revealed that P2P values were significantly higher at both 10 PT (*p* = .004) and 25% MVC (*p* = .005). Similar to NVS, P2P STA latency was not affected by background EMG level (*F*_1,8_ = 0.444, *p* = .524) or stimulus amplitude (*F*_1,8_ = 0.005, *p* = .944), and there was no significant interaction between these factors (*F*_1,8_ = 1.104, *p* = .324). For comparison, the bottom row of bar graphs in Figure 4 depicts the group data for P2P STA amplitude and latency across the different test conditions.

## 4 DISCUSSION

Our results support NVS as a viable approach for assessing cutaneomotor reflex function. Plantar stimulation evoked later muscle responses than those observed recently using noisy tendon vibration to engage faster muscle stretch reflex circuitry (Mildren et al., 2017, 2019). The muscle response latencies observed in this study (96.3 ms for NVS; 93 ms for pulses) suggest plantar stimulation engaged primarily cutaneomotor reflex circuitry, which, in comparison to muscle stretch reflex circuitry, has slower afferent conduction velocities (Kakuda, 1992), and an added synaptic delay due to its polysynaptic nature (Aniss et al., 1992).

### 4.1 The effect of increasing stimulus amplitude

Increasing stimulus amplitude had a facilitatory effect on cutaneomotor responses, presumably by increasing cutaneous afferent firing rates (Strzalkowski et al., 2017). A larger cutaneous stimulus would result in greater cutaneous input to the motor neuron pool of TA, and therefore, stronger cutaneomotor coupling. Depending on whether the responses were normalized to the background EMG level or not, two distinct results were obtained, offering an important insight into cutaneomotor reflex function. If we take the approach of previous studies and do not normalize the responses to background EMG level (Duysens et al., 2008; Forth & Layne, 2007), we find that muscle responses increase with higher voluntary background contractions for a fixed stimulus amplitude. However, using normalization procedures that effectively isolate the proportion of the muscle responses attributable to the stimulus input from the voluntary component of the response (Arntz et al., 2019), we found that increasing voluntary background EMG either had no effect on (discrete pulses) or significantly decreased (continuous NVS) the proportion of the response attributable to the input stimulus. These results demonstrate an important trade‐off in cutaneomotor reflex responses when two distinct inputs to the motor neuron pool—namely, cutaneous afferent input and voluntary descending input—compete to determine spinal outflow through a final common pathway.

### 4.2 The effect of increasing background EMG

Our results agree with previous literature: we observed increases in non‐normalized cutaneomotor responses when the background level of contraction was raised from 12.5%–25% MVC. Forth & Layne (2007) compared responses evoked from discrete tap stimuli delivered to the foot sole (square wave, 100‐ms duration, 3 mm indentation), while participants held a 0%, 40%, or 80% MVC background level of contraction in soleus and lateral gastrocnemius, observing a linear increase in response amplitudes. Similarly, Duysens et al. (2008) applied mechanical vibration at 90, 110, and 130 Hz to the foot sole (sine wave, 250‐ms duration, undefined amplitude), while participants held a 0%, 5%, or 10% background level of contraction in gastrocnemius, tibialis anterior, biceps femoris, and rectus femoris; again, these researchers found a linear increase in cutaneomotor response amplitude with increasing levels of background activity. However, by normalizing the level of background EMG, here we show that the proportion of the response attributable to cutaneous stimulation either stays the same or decreases when voluntary descending input to the motor neuron pool increases. This trade‐off, which has recently been explored and exploited to study mechanisms of vestibular balance control (Arntz et al., 2019), illustrates that the capacity for cutaneous stimulation to drive muscle responses does not increase with higher levels of background muscle activation, and may, in fact, diminish.

### 4.3 The timing of cutaneomotor responses

On average, we found a peak cutaneomotor response latency of 93 ms using NVS. This value is in general agreement with previous reports of cutaneomotor responses occurring around 50 ms and oscillating for 30–40 ms (Duysens et al., 2008; Forth & Layne, 2007) or longer (Aniss et al., 1992). In contrast, using noisy tendon vibration and cutaneous anesthesia, Mildren et al. (2017, 2019) observed monosynaptic stretch reflexes occurring at shorter latencies of ~35–40 ms in the lower‐limb. As can be seen in the cross‐covariance plots of the present study (see Figure 3), while the peak of the response is occurring later, there are smaller oscillating peaks occurring as early as 50 ms. One likely interpretation of this result is that our plantar stimulation resulted in mechanical spreading to non‐cutaneous afferent receptors, such as muscle spindle primary endings, which would manifest as correlated muscle activity at shorter, monosynaptic latencies.

### 4.4 The relative contribution of different classes of cutaneous afferents

Without supporting human microneurographic recordings from the different cutaneous afferent classes, it is difficult to ascertain the precise contribution of each class to the overall cutaneomotor response. However, previous observations hint toward an important role for FAI afferents in driving plantar cutaneous reflexes. Fallon et al. (2005) provided evidence for FAI afferents having the strongest synaptic coupling to lower‐limb motor units. Additionally, the strongest coherence observed in the present study lies directly in the zone of maximal sensitivity for FAI afferents (Bolanowski et al., 1988; Johansson & Vallbo, 1979; Johnson, 2001; Johnson et al., 2000; Kennedy & Inglis, 2002; Löfvenberg & Johansson, 1984; Strzalkowski et al., 2017). Therefore, FAI afferents are ideally suited to encode the stimulus frequencies that get transmitted into lower motor neuron activity. Different bandwidths of NVS (e.g., including higher frequencies) could potentially reveal inter‐receptor class differences in cutaneomotor responses; however, pilot data were not promising in this regard. It should be noted that previous work using 250 Hz sinusoidal vibrations (Peters et al., 2016) and pilot testing for this study (which also involved a 0–300 Hz NVS waveform) indicates that cutaneomotor coupling is non‐existent, or at best, much less clear if higher frequency (Pacinian‐range) vibrations are included in the input stimulus.

We emphasize that NVS is a tool for measuring and monitoring cutaneous reflexes; however, functionally, cutaneous reflexes in the lower limb that are triggered and scaled by FAI input could serve an important purpose for balance control. FAI afferents are known to be sensitive to “microslip”—that is, microscopic slippage events between the skin and a contacting surface—scaling their responses to the slipperiness of the texture and slip velocity (Johannson & Westling, 1984; Macefield et al., 1996; Srinivasan et al., 1990). FAI afferents also have the highest innervation density of the four cutaneous receptor types in the plantar foot sole (Kennedy & Inglis, 2002; Strazalkowski, Peters, Inglis, & Bent, 2018). Taken together, slippage events could be detected and acted upon reflexively to maintain balance through strong cutaneomotor coupling to FAI afferents in the lower limb. Such a neural mechanism is likely important for responding to perturbations and maintaining balance, particularly in slippery situations (e.g., icy sidewalks, soapy bathtubs, etc.).

### 4.5 Future directions

However, at this time, we cannot rule out concomitant influence from the activation of other somatosensory receptors by the spread of mechanical vibration through tissue, particularly to intrinsic foot muscle spindles. Future human microneurography will be needed to unravel the relative contribution of the different cutaneous receptor classes in shaping cutaneomotor reflexes. In addition to providing a powerful tool for non‐invasively investigating spectrotemporal aspects of human cutaneomotor coupling, supra‐threshold NVS may also have several therapeutic applications that remain to be investigated. For example, NVS stimulation could potentially be used to counteract muscle atrophy or weakness which commonly occurs in long‐duration space flight and bed‐rest patients (Forth & Layne, 2007; Colombo, Wirz, & Dietz, 1998; Layne, Mulavara, Pruett, et al., 1998; Layne, Mulavara, McDonald, et al., 2000; Layne, Forth, Baxter, & Houser, 2002). NVS‐based cutaneous reflex assessments could also be performed by vibration‐emitting wearable technologies, complimenting other therapeutic interventions aimed at enhancing cutaneomotor reflexes and standing balance function (Priplata, Niemi, Harry, Lipsitz, & Collins, 2003; Jenkins et al., 2009; Lipsitz et al., 2015).

## CONFLICT OF INTEREST

The authors have none to declare.

## AUTHOR CONTRIBUTIONS

All authors took part in the experiment design, data collection/interpretation, and editing of the manuscript. R.M.P. additionally analyzed the data, created the figures, and wrote the first draft of the manuscript.
